# Comparative Study of Biochar from Different Biomass Feedstocks: Toward Sustainable Resource Utilization and Environmental Applications

**DOI:** 10.3390/molecules31010037

**Published:** 2025-12-22

**Authors:** Nina Đukanović, Tamara Apostolović, Jasmina Anojčić, Sanja Mutić, Tijana Marjanović Srebro, Gábor Kozma, Cora Deák, Snežana Maletić, Jelena Beljin

**Affiliations:** 1 University of Novi Sad, Faculty of Sciences, Department of Chemistry, Biochemistry and Environmental Protection, Trg Dositeja Obradovića 3, 21000 Novi Sad, Serbiajasmina.anojcic@dh.uns.ac.rs (J.A.);; 2 Interdisciplinary Excellence Centre, Department of Applied and Environmental Chemistry, University of Szeged, Rerrich Béla Tér 1, 6720 Szeged, Hungary

**Keywords:** biochar, pyrolysis, hardwood, corn cob, wheat straw

## Abstract

This study examines the structural, chemical, and thermal properties of biochars from slow pyrolysis of hardwood (HW), corn cob (CC), and wheat straw (WS) at 400 °C and 700 °C, evaluating their potential in environmental and industrial applications. A combination of spectroscopic, crystallographic, thermal, and microscopic techniques was employed to monitor the degradation of biomass components and the development of the carbonaceous matrix. The results show that pyrolysis temperature has a significant impact on the properties of biochar. Higher temperatures (700 °C) increased the pH (up to 10.3 for WS700), the carbon content (e.g., 89.8% for HW700), the ash content (up to 24.8% for WS700), and the specific surface area (e.g., 306.87 m^2^/g for CC700) while decreasing polar functional groups and volatile matter (as confirmed by FTIR). SEM showed enhanced porosity at 700 °C, which was supported by BET analysis. XRD and Raman showed increased graphitization and structural order with temperature, especially for HW and CC biochars, while WS biochars retained mineral components like SiO_2_ and CaCO_3_. TGA analysis showed improved thermal stability at 700 °C only for biochar derived from wheat straw, while HW and CC biochars showed similar total mass loss regardless of pyrolysis temperature. These biochars exhibit high potential for soil remediation (high pH), water purification (large surface area), and carbon storage (high aromaticity), with HW700 and CC700 also suitable for high-temperature industrial applications due to their stability.

## 1. Introduction

Biochar is a carbon-rich material derived from biomass through high-temperature pyrolysis, a process that thermochemically decomposes organic matter in the absence of oxygen [1]. The physicochemical properties of the resulting material—including carbon content, porosity, aromaticity, and mineral phases—depend strongly on pyrolysis temperature, heating rate, residence time, and feedstock composition [2,3].

Due to its structural tunability and stability, biochar has attracted increasing attention for environmental and industrial applications, including adsorption of heavy metals and organic pollutants [4,5], soil improvement and water retention [6], carbon sequestration [7], and the reduction of greenhouse gas emissions [8]. Using agricultural and forestry residues as biochar feedstocks also supports sustainable waste management and circular-economy principles [9].

At the global scale, agricultural activities generate vast quantities of lignocellulosic residues, and their accumulation is expected to increase further with rising food production demands [10,11]. This highlights the need for sustainable biomass valorization strategies, particularly in predominantly agricultural regions. The Autonomous Province of Vojvodina (Serbia), with extensive areas of arable land and forest resources (approximately 1.78 million hectares of arable land and around 150,000 hectares of forested area), produces significant amounts of crop residues and woody biomass annually [12,13]. While part of this material is reused (e.g., as animal feed or construction material), large quantities remain underutilized or are commonly managed through open burning practices, known to release greenhouse gases, reduce soil fertility, and contribute to air pollution [14]. Converting such residues into biochar therefore represents a promising and locally relevant approach for waste reduction, environmental protection, and carbon stabilization.

Lignocellulosic biomass is among the most widely used feedstocks for biochar production due to its abundance, low cost, and structural diversity [15]. Its main constituents—cellulose, hemicellulose, and lignin—decompose differently during thermal conversion, shaping the evolution of functional groups, porosity, ash composition, and aromatic carbon structures in the resulting biochar [16,17]. Although numerous studies have examined biochars derived from individual feedstocks or single pyrolysis temperatures, systematic cross-feedstock comparisons conducted under identical pyrolysis conditions remain limited. Moreover, many studies focus on isolated properties, such as surface area or functional groups, without integrating structural, morphological, thermal, and chemical characterization within a unified framework. This limits a comprehensive understanding of how biomass composition and pyrolysis conditions jointly control biochar properties and performance, as well as its suitability for specific environmental or industrial applications.

To address these limitations, this study provides a detailed comparative characterization of biochars produced from hardwood, corn cob, and wheat straw at two pyrolysis temperatures (400 °C and 700 °C). By combining FTIR, Raman spectroscopy, XRD, BET surface area analysis, SEM/EDS, thermogravimetric analysis, and elemental composition, the work offers a comprehensive assessment of structural, chemical, and thermal transformations. This approach advances current knowledge by establishing direct links between feedstock composition, pyrolysis conditions, and the resulting biochar properties, thereby extending the present state of the art and supporting rational, application-oriented biochar selection. The specific objectives were to (i) evaluate the effects of different feedstocks and pyrolysis temperatures on the chemical, structural, and thermal characteristics of the produced biochars using a multi-technique approach, and (ii) assess how these properties influence their suitability for various environmental applications.

## 2. Results and Discussion

### 2.1. Proximate and Ultimate Analysis

Table 1 summarizes the impact of pyrolysis temperature and different feedstock biomass on various physicochemical properties of biochars.

All biochars exhibited alkaline pH, and pyrolysis temperature consistently increased alkalinity across all feedstocks. This trend reflects the progressive loss of acidic surface functional groups and the enrichment of alkaline mineral phases at higher temperatures, as is commonly reported for lignocellulosic biochars [18,19].

Based on previous research, the relative proportions of cellulose, hemicellulose, and lignin differ substantially among hardwood, corn cob, and wheat straw. Hardwood generally contains a higher proportion of lignin, while wheat straw and corn cob are richer in cellulose and hemicellulose [20,21,22]. These differences strongly influence devolatilization behavior and carbon structure development during pyrolysis.

Pyrolysis temperature strongly influenced ash concentration, with higher temperatures producing more mineral-rich residues as organic matter volatilized. Feedstock type also played a key role: agricultural residues generated biochars with substantially higher ash content than hardwood, reflecting their naturally higher inorganic fraction. This compositional difference aligns with the established view that low-ash hardwood biochars generally possess higher carbon purity and are better suited for applications requiring minimal mineral interference [23].

The increase in fixed carbon content is a direct result of the devolatilization process, which is directly related to the reduction in the amount of volatile matter [24]. Hardwood biomass has a relatively high lignin content, which contributes to a higher fixed carbon content in biochar [25]. At a temperature of 400 °C, a significant part of the volatile components remains in the biochar, because the pyrolysis process did not completely remove all organic volatile components. The volatile matter content in all biochars decreased after pyrolysis at 700 °C, primarily due to the dehydration of hydroxyl groups, the evaporation of light compounds during pyrolysis, and structural transformations in cellulose and lignin [26]. These trends are in good agreement with previous studies on lignocellulosic biochars, which also report higher fixed carbon contents and lower volatile matter at elevated temperatures (typically 600–800 °C) [24,25,26]. Such changes are generally associated with improved thermal stability and a higher degree of carbonization.

Table 1 also shows the results of elemental CHNS analysis. In the case of all biochars, the carbon content increased with increasing pyrolysis temperature (in the case of biochar obtained from wheat straw, there was a slight difference in carbon between the two pyrolysis temperatures). The increase in C content due to increasing pyrolysis temperature is a consequence of dehydration, i.e., faster release of O and H, as well as the loss of functional groups such as carboxyl and hydroxyl, which are associated with these atoms [27]. The hydrogen content increased with pyrolysis temperature in the biochar obtained from hardwood, while in the other two types of biochar, the H content decreased with increasing pyrolysis temperature. This behavior can be explained by the lignin-rich nature of hardwood biomass, as lignin decomposes gradually over a broad temperature range and promotes the formation of condensed aromatic carbon structures during pyrolysis, which may contain thermally stable hydrogen [28,29]. The N content of all biochars was lower at higher pyrolysis temperatures, which can be attributed to the volatilization of nutrients at high temperature that were resistant to low temperature and not easily volatile [30], confirming previous research thinking that pyrolysis leads to a decrease in nitrogen content in biochar [29]. Sulfur in the hardwood biochars was below the detection limit. The differing trends in sulfur content in WS and CC biochar with increasing pyrolysis temperature can be explained by the chemical nature of sulfur in the raw materials. Many studies, such as those by Cantrell et al. [31], Al-Wabel et al. [32], and Zhao et al. [33], confirm that sulfur generally decreases with temperature due to the volatilization of sulfur-containing compounds, which aligns with the behavior of WS biochar, where sulfur, mainly in the form of sulfates, becomes volatile and is lost at higher temperatures. Zornoza et al. [34] observed the opposite trend in cotton residue biochar, where sulfur content increased with temperature due to the presence of more thermally stable organic sulfur compounds and the formation of mineral sulfates. This aligns with CC biochar, where sulfur remains trapped in the carbon structure as stable heteroaromatic compounds, such as thiophenes. Research by Cheah et al. [35] has shown that the primary factor determining the total sulfur content in biochar is the type of feedstock, while temperature and the thermal conversion method are crucial for sulfur speciation.

The H/C ratio serves as an indicator of biochar aromaticity, while the O/C and (O + N)/C ratios provide insights into the polarity of biochar [31]. The calculated molar ratios of H/C for all obtained biochars were <1.5, indicating the presence of highly unsaturated and aromatic structures [34].

The O/C molar ratios were equally low for all biochars, indicating a very low polarity of organic matter (hydrophobicity) and a low content of oxygen functional groups [36]. For biochars with an O/C ratio of less than 0.2, half-lives of more than 1000 years are predicted. For an O/C 0.2–0.6 biochar sample, the half-life would be 100–1000 years, and for O/C > 0.6, the half-life would be <100 years [37]. Based on this observation, all six biochars are predicted to have a half-life exceeding 1000 years.

The (O + N)/C ratio is a key parameter for assessing the chemical properties and potential applications of biochars. It reflects the material’s polarity, hydrophilicity, and water retention capacity, which are crucial factors in fields like soil enhancement, water purification, and carbon sequestration. For all three feedstocks, biochar pyrolyzed at a lower temperature have a higher (O + N)/C ratio. A higher (O + N)/C ratio indicates a greater presence of polar functional groups, such as hydroxyl (-OH), carboxyl (-COOH), and nitrogen-containing groups, which enhance the biochar’s hydrophilicity and chemical reactivity. In contrast, a lower (O + N)/C ratio suggests a more aromatic and hydrophobic structure, making the biochar more suitable for long-term carbon storage and other applications requiring high stability and reduced reactivity [1,38]. Given that O/C and (O + N)/C decreased with increasing temperature, this indicates the loss of some functionalities of oxygen and nitrogen. Comparable O/C and H/C ranges have been reported for hardwood and crop-residue-derived biochars produced at similar temperatures, confirming that the materials obtained at 700 °C fall within the stability domain typically associated with long-lived, highly aromatic biochars [37]. By contrast, the higher (O + N)/C ratios observed at 400 °C indicate a greater abundance of polar functional groups and a more reactive surface, which can be advantageous for applications such as sorption of polar contaminants or soil amendment.

The estimated higher heating values (HHV) of the biochars ranged from approximately 23 to 31 MJ kg^−1^, indicating that all six materials possess energy densities comparable to typical lignocellulosic biochars. For each feedstock, HHV was slightly higher at 700 °C, which is consistent with the increase in carbon content, fixed carbon fraction, and the concurrent decrease in oxygen content at higher pyrolysis temperature. Biochars derived from hardwood and corn cob showed similar HHV values, while wheat straw biochars (WS400) exhibited somewhat lower HHV, reflecting their higher ash content and lower carbon fraction. Overall, these trends are in line with the notion that progressive carbonization and devolatilization at higher temperatures lead to a moderate improvement in the fuel quality of biochar [39].

The observed trends in physicochemical properties are primarily governed by the sequential thermal degradation of lignocellulosic components. Hemicellulose decomposes at relatively low temperatures, contributing to volatile release and oxygen-containing functional groups, while cellulose degradation promotes pore development and partial aromatization. Lignin decomposes over a broader temperature range and is responsible for the formation of condensed aromatic carbon structures. Consequently, increasing pyrolysis temperature leads to carbon enrichment, ash concentration, and progressive aromatization, as reflected by decreasing H/C and O/C ratios, reduced surface functionality, and enhanced thermal stability.

### 2.2. Fourier Transform Infrared Spectroscopy (FTIR)

**Figure 1:**
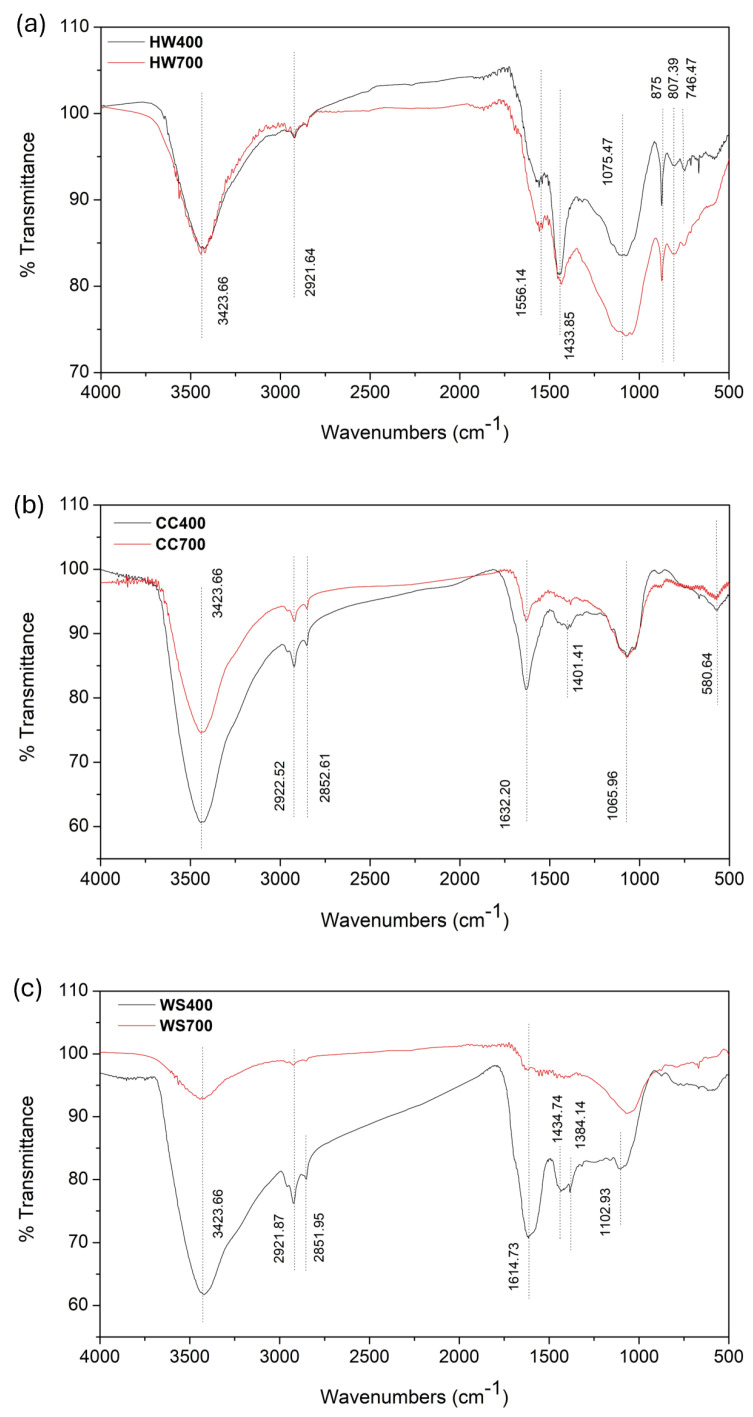
FTIR spectra of the (**a**) hardwood biochar pyrolyzed at 400 and 700 °C, (**b**) corn cob biochar pyrolyzed at 400 and 700 °C, and (**c**) wheat straw biochar pyrolyzed at 400 and 700 °C.

The FTIR spectra of all six biochars are shown in Figure 1a–c. With FTIR analyses, the bonds between carbon, hydrogen, and oxygen in the biochar can be determined, and insights can be gained about the suitability of the raw material for energy production [40]. The evolution of FTIR spectra with increasing pyrolysis temperature reflects the progressive decomposition of lignocellulosic components. Hemicellulose and cellulose, which are rich in oxygen-containing functional groups, undergo extensive dehydration and depolymerization at elevated temperatures, while lignin-derived aromatic structures persist, leading to increased chemical stability and surface aromatization. For all three different feedstocks, it is evident that at higher pyrolysis temperatures, many functional groups disappear, and the FTIR spectra become simpler and flatter, indicating that the active chemical structures on the surface decompose and the biochars become more chemically stable due to increased carbonization and removal of volatiles [41].

For all six biochars, peaks are dominantly concentrated in the ranges of 3423–2851 cm^−1^ and 1614–746 cm^−1^, indicating that they possess similar functional group structures. Despite overall similarity in the types of functional groups, the degree of change with temperature differs significantly between the biochars depending on the feedstock. This highlights the impact of the original biomass composition, particularly lignin, cellulose, and hemicellulose content, on the final biochar properties. For example, the WS and CC biochars show a more significant reduction in hydroxyl (O–H) and aliphatic (C–H) groups with temperature compared to the HW biochar. This behavior is consistent with the higher cellulose and hemicellulose content of wheat straw and corn cob [21,22], which decompose more readily at lower temperatures. In contrast, HW biochar retains more pronounced aromatic and lignin-related features, even at 700 °C, reflecting the higher thermal stability and slower decomposition of lignin-rich biomass.

The characteristic peak at 3423 cm^−1^ in the FTIR spectrum is usually attributed to the stretching vibration of hydroxyl groups (O-H) involved in hydrogen bonding [42], which decreases at higher temperatures due to the loss of hydrogen and oxygen [43]. At high pyrolysis temperature, gradual aromatization of biochar occurs, resulting in increased hydrophobicity and a decrease in the O-H absorption peak [44]. The O-H bonds in these materials originate from hydroxyl functional groups present in their basic components and inter-structural units of lignocellulosic biomass [15,42] or in residual water [38]. A significant decrease in the peak at 3423 cm^−1^ is observed in the WS700 biochar compared to WS400, indicating the removal of hydroxyl groups and the reduction of polar components. This reduction in polar functionalities contributes to increased hydrophobicity and chemical stability, which is desirable in applications such as carbon sequestration or pollutant adsorption.

The peaks around 2921–2851 cm^−1^ are associated with the stretching vibration of the C-H bond and -CH_2_ groups, i.e., aliphatic groups, originating from compounds in the biomass with C-H chains, such as fatty acids and fatty alcohols [41,42]. The pronounced reduction of O–H and aliphatic C–H bands at higher temperatures indicates extensive dehydration reactions and the breakdown of polysaccharide chains, which are primarily associated with cellulose and hemicellulose degradation.

The peaks around 1434–1401 cm^−1^ are also associated with the stretching vibration of C–H in aliphatic carbon-hydrogen chains in biomass, particularly related to methyl (CH_3_) functionalities [42]. The peak intensities for all three biomasses weaken at higher temperatures, indicating stabilization of the biochars due to the degradation of aliphatic structures [44].

The peak at 1384 cm^−1^ corresponds to the asymmetric and symmetric C-O vibrational bands of the carboxylic acid group on the biochar surface [45] and was recorded only in WS400. According to the study by Liu et al. [44], the absorption peak at around 1350 cm^−1^ indicates the presence of N=O in biochar, which originates from amino acids and fatty substances, while Liu et al. [46] claim that the peak at 1384 cm^−1^ represents C=O stretching vibrations in the carboxyl group and -OH bending of phenol.

The 1000–1800 cm^−1^ range was attributed mainly to oxygen-containing functional groups [47]. The band peaks in the range of 1632–1556 cm^−1^ probably originate from the stretching C=C vibrations of aromatic rings [41,42], but they could also be based on the stretching C=O vibrations of quinone groups, or the bending O-H vibrations of physiosorbed water molecules, or be the result of the absorption of atmospheric CO_2_ [48]. High pyrolysis temperatures increasing the degree of aromatization of biochar is also confirmed by the fact that the C=C peak is more pronounced at higher pyrolysis temperatures [44].

Peaks in the range 1102–1065 cm^−1^ were also observed in the FTIR spectrum. These peaks are associated with the C–O–C stretching vibration (ether bond) in polysaccharide compounds such as cellulose and hemicellulose [42]. The intensity of peak 1102 cm^−1^ decreases significantly in WS700 and CC700, indicating the decomposition of oxygen-containing polysaccharides with increasing temperature. This peak is less affected in HW biochar, aligning with its lower polysaccharide content and greater lignin proportion.

Peaks at less than 900 cm^−1^ in FTIR spectra usually originate from vibrations of C-H bonds in aromatic units and bending vibrations of C-H, C-C bonds in ring structures of lignin, C–H or N–H out-of-plane bending, as well as carbon skeletons in biochar. In that low-frequency range, vibrations associated with aromatic structures and deformations of alkane and aromatic compounds dominate. At lower pyrolysis temperatures (400 °C), these peaks are more pronounced because lignin is not completely decomposed. At higher temperatures (700 °C), the peaks are reduced or completely disappear due to further aromatization and the formation of a stable carbon skeleton [45,49]. These peaks are significantly more pronounced in the FTIR spectra of biochars obtained from hardwood, which is expected given the higher lignin content in hardwood compared to wheat straw and corn cob [20,21,22]. The persistence of these low-frequency aromatic vibrations, particularly in hardwood biochars, further confirms the formation of a stable lignin-derived aromatic carbon skeleton at higher pyrolysis temperatures.

The differences in FTIR spectra between the biochars reflect the inherent variability in feedstock composition and pyrolysis behavior. The presence or loss of specific functional groups not only informs us of chemical stability but also directly relates to potential applications. WS and CC biochars, with more labile components, undergo significant structural simplification at high temperatures, making them suitable for short-term soil applications or nutrient release. HW biochars, due to their more stable aromatic structure, are more suitable for long-term applications like carbon sequestration or use in water treatment, where durability and stability are key.

### 2.3. Scanning Electron Microscopy (SEM), Specific Surface Area (SSA), and EDS Observations

**Figure 2:**
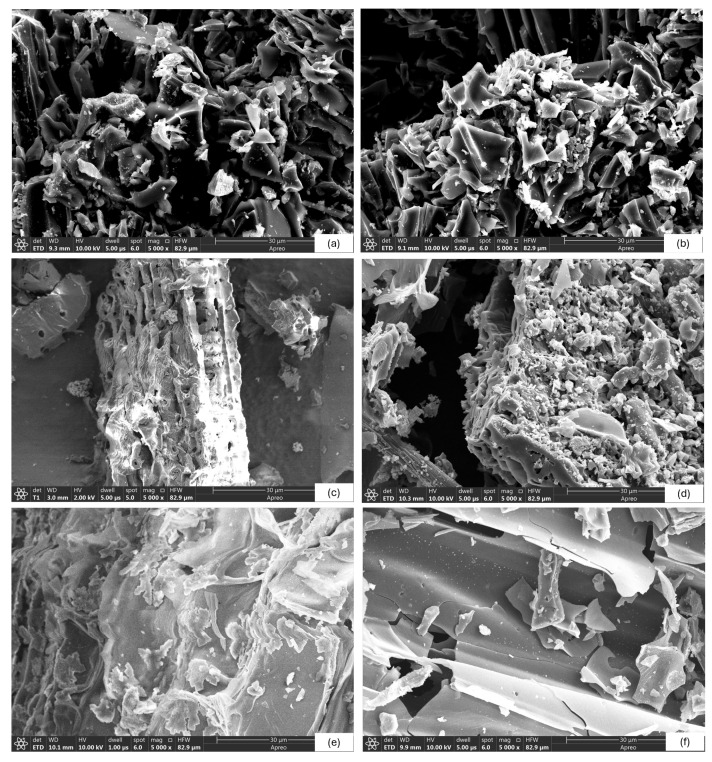
SEM images of biochar: (**a**) HW400, (**b**) HW700, (**c**) CC400, (**d**) CC700, (**e**) WS400, (**f**) WS700.

SEM images of biochars at a magnification of 5000 are shown in Figure 2. The observed morphological differences among biochars are primarily governed by feedstock composition and pyrolysis temperature. The formation and evolution of pore structures are closely linked to the release of volatile compounds and the collapse of organic matrices during the thermal degradation of lignocellulosic components. Visual inspection of images exemplifies the differences in irregular and distinct porous surfaces. It is a known fact that the pyrolysis temperature plays a vital role in biochar properties, and different raw materials show different morphological characteristics [50]. At lower temperatures (300–400 °C), biochar retains more of the original biomass structure, with less developed porosity. At higher temperatures (500–700 °C), more intensive decomposition of organic components occurs, which results in increased porosity and the formation of micropores and mesopores (the surface structure was seriously damaged and formed an obvious flaky structure when the pyrolysis temperature exceeded 500 °C [51]).

Accordingly, the biochars produced at high pyrolysis temperature (i.e., HW700, CC700, and WS700) show larger porosity than biochar samples produced at low pyrolysis temperatures (Figure 2b,d,f). The surface morphology of the samples (b, d, f) had gradual pore opening with deep holes, while the surface morphologies of the samples (a, c, e) were relatively flat and simple. At elevated temperatures, extensive devolatilization and carbon skeleton rearrangement promote the development of interconnected micro- and mesoporous structures.

Detailed structural analysis of individual biochars indicates that the structure of HW400 (Figure 2a) is relatively porous, with preserved remnants of the original plant microstructure and the presence of macropores. Cellulose and hemicellulose are partially degraded, while lignin still plays a key role in maintaining structural stability. Biochar HW700 (Figure 2b) has a significantly higher void ratio compared to HW400. Increased structural fragmentation is observed, which is a consequence of more intense pyrolysis. The surface of the biochar shows a larger number of small pores that are a consequence of the decomposition of cellulose, hemicellulose, and partial degradation of lignin. Higher temperature led to the removal of volatile substances and the creation of a more stable carbon skeleton. CC400 biochar (Figure 2c) has a relatively smooth structure with less pronounced porosity compared to hardwood biochar, which indicates that at this temperature, the chemical bonds and lignocellulosic components have not been completely decomposed. At a pyrolysis temperature of 700 °C (Figure 2d), the porosity significantly increased compared to CC400. Micro- and mesopores are present, which indicates almost complete decomposition of lignocellulosic fibers. WS400 biochar (Figure 2e) exhibits a fibrous structure, with surface cracks and limited porosity. Compared to WS400, WS700 (Figure 2f) has significantly more cracks, deep and wide pores of small size, and almost completely decomposed fibers. These differences highlight the dominant role of lignocellulosic composition in pore development. Hardwood biochars benefit from the gradual degradation of lignin, which preserves structural integrity while enabling progressive pore formation. In contrast, wheat straw and corn cob, rich in cellulose and hemicellulose, undergo faster matrix collapse, leading to rapid pore opening at high temperatures but less structural stability at lower pyrolysis temperatures.

The SEM results of the biochar are confirmed by the specific surface areas (SSAs) obtained by BET analysis, which are 176.53 m^2^g^−1^, 283.82 m^2^g^−1^, 20.87 m^2^g^−1^, 306.87 m^2^g^−1^, 3.68 m^2^g^−1^, and 80.88 m^2^g^−1^ for HW400, HW700, CC400, CC700, WS400, and WS700, respectively. The BET results are consistent with SEM observations, confirming that pore development and surface area enhancement are driven by temperature-dependent devolatilization and feedstock-specific degradation pathways. Hardwood biochar retains a high specific surface area, even at a lower temperature (400 °C), but a higher temperature (700 °C) further improves porosity and specific surface area. This is the result of the slow decay of the lignin component of the wood. Cob corn biochar shows a drastic jump in porosity and specific surface area at 700 °C, which indicates rapid degradation of cellulose and hemicellulose with the formation of micropores. Wheat straw biochar has the lowest specific surface area at 400 °C and weaker pore development at 700 °C compared to other materials. This is probably due to its chemical composition and faster degradation at lower temperatures, which may result in a less stable porous structure.

The results of the study are consistent with previous studies, i.e., Brown et al. [52], Singh et al. [53], Wang et al. [54], Kaur et al. [15], Yargicoglu et al. [55], Chaves Fernandes et al. [27], and Chatterjee et al. [30], in which scientists, based on the characterization of biochars obtained from different biomasses, concluded that with increasing temperature, a more porous structure and a higher SSA were obtained. In particular, the specific surface areas obtained for CC700 and HW700 (306.87 and 283.82 m^2^g^−1^, respectively) lie within or above the range commonly reported for high-temperature biochars from similar feedstocks [52,53,54,55], indicating that the 700 °C treatment is more favorable for generating well-developed pore structures. In contrast, the much lower SSA values at 400 °C, especially for WS400, reflect incomplete pore development and are more comparable to low-temperature biochars reported in the literature [27,28].

Elemental analysis of biochar (EDS), combined with CHNS elemental analysis, showed that in all biochars, the carbon content was significantly higher compared to the other elemental compositions, while the second main element was oxygen, also in significant amounts (detailed values of the contents of C and O are shown in Table 1). Other element contents (Mg, Al, P, Cl, Mn, Cu, S, etc.) in biochars were mostly less than 1%, except Ca (1–2%) and Si (more than 1%).

### 2.4. Thermal Stability Analysis by TGA-dTG

To understand the degradation behavior and suitability of biochar for high-temperature applications, it is necessary to analyze the thermal stability of biochar. This thermoanalytical technique provides insight into the thermal degradation of biochar, i.e., it monitors the weight loss and/or gain of the sample as a function of time or temperature [38]. Specifically, the TG curve (weight %) shows the change in the mass of the sample during heating as a function of temperature, and the weight loss indicates the degradation of different organic components. Differential thermogravimetry (dTG) is the first derivative of the TG function [56], and the peaks in the dTG curve represent the temperatures at which the highest rate of mass loss occurs, which usually indicates the degradation of specific organic components (expressed as %/°C).

**Figure 3:**
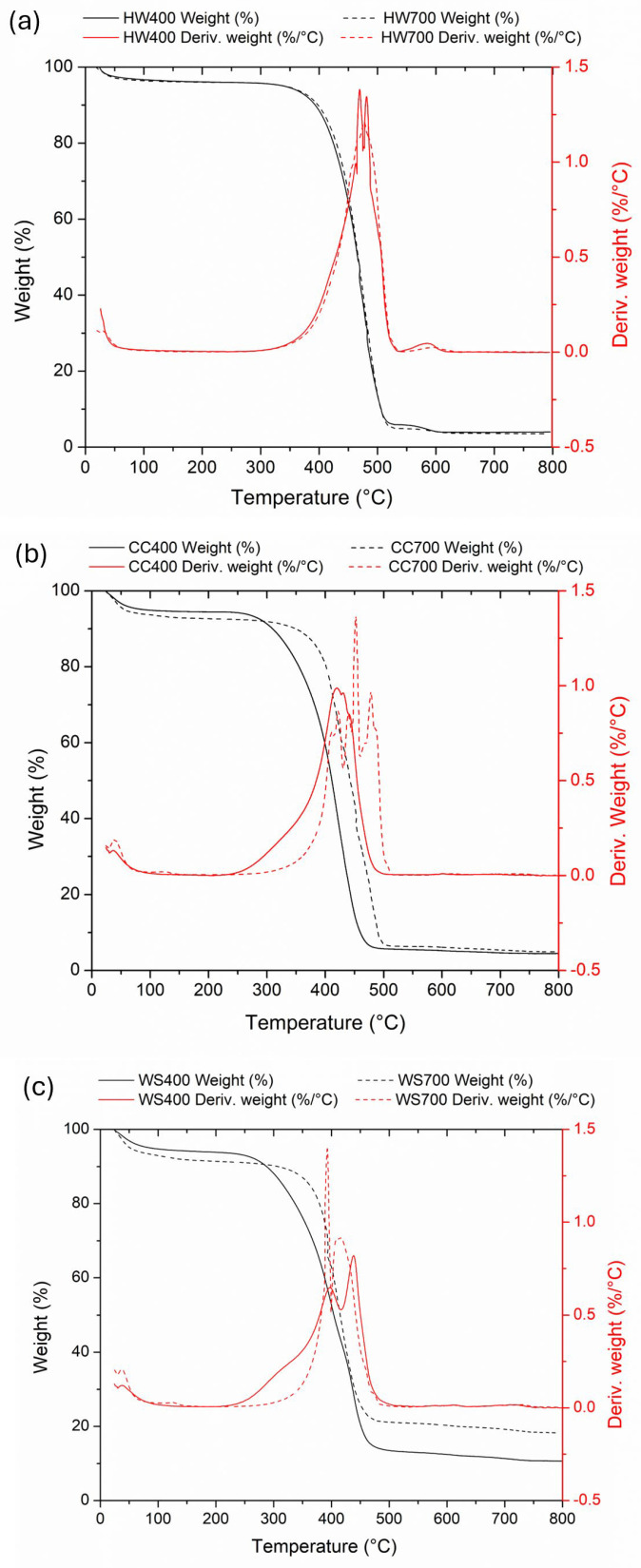
TGA and dTG analysis of (**a**) hardwood biochar pyrolyzed at 400 and 700 °C, (**b**) corncob biochar pyrolyzed at 400 and 700 °C, and (**c**) wheat straw biochar pyrolyzed at 400 and 700 °C.

Figure 3 displays the TGA and dTG thermographs against temperature at a heating rate of 5 °C min^−1^. TG-DTG curves for biochar pyrolyzed at 400 °C are shown as solid lines, and those for biochar pyrolyzed at 700 °C as dashed lines. TG data (weight %) are given as black lines and DTG (first derivative of weight %/°C) as red lines.

The area under the TG/dTG curve, based on the facts explained in their works by De la Rosa et al. [57], Merino et al. [58], and Merino et al. [59], is divided into five parts, which represent different degrees of resistance to thermal oxidation: **moisture** (20–105 °C)—free water that is not chemically bound to the material structure; **moisture and very labile organic matter (OM)** (105–200 °C)—residual water that is more tightly bound to the pores and surface of the sample, as well as small molecules (hydrocarbons, fatty acids, waxes); **labile OM** (200–400 °C)—mainly polysaccharides; **intermediate OM** (400–600 °C)—proteins and aliphatic compounds; and **recalcitrant OM** (600–800 °C)—lignins, polyphenols, and condensed aromatic structures. Table 2 shows the cause of the weight loss of biochar at certain temperature ranges for TG-dTG, as well as the percentage of weight loss at certain temperature ranges.

Based on the data presented in Table 2 and the TG/dTG curves shown in Figure 3, the total weight loss for all biochars in the temperature range of 20 °C to 800 °C varies between 81.8% and 96.5%. The biggest weight loss for biochars HW400 and HW700 occurs between 400 °C and 500 °C and is about 85%, while for the other four biochars, this interval is somewhat wider, from 300 °C to 500 °C. The precious weight loss in these temperature intervals (200–400 °C and 400–600 °C) is presented in Table 2.

An analysis of the TG–DTG data indicates that the effect of increasing pyrolysis temperature on thermal stability depends strongly on the feedstock type, as also reported for lignocellulosic biochars in previous studies [57,58,59]. For hardwood and corn cob-derived biochars, the total mass loss of between 20 and 800 °C remains comparable between samples produced at 400 and 700 °C (HW: 96.0–96.5%; CC: 95.1–95.5%), suggesting that a higher pyrolysis temperature does not significantly alter the overall extent of thermal degradation. However, differences are evident in the degradation profiles: biochars produced at 700 °C exhibit a shift of the main decomposition events toward higher temperatures and a greater proportion of mass loss occurring within the intermediate and recalcitrant organic matter regions (400–800 °C), reflecting increased carbonization and structural reorganization [57,58,59].

In contrast, wheat straw-derived biochars show a pronounced decrease in total mass loss with increasing pyrolysis temperature (from 89.3% for WS400 to 81.8% for WS700), indicating enhanced thermal stability at higher temperatures. This behavior can be attributed to the higher mineral content and lower lignin proportion of wheat straw biomass [21], which promote ash enrichment and the formation of more thermally resistant inorganic–organic matrices at elevated pyrolysis temperatures.

The TG and dTG curves mutually confirm the conclusions reached, because at the temperature range where the TG curve established the greatest weight loss, a peak appeared on the dTG curve, confirming the cause of the weight loss.

### 2.5. XRD Analysis

**Figure 4:**
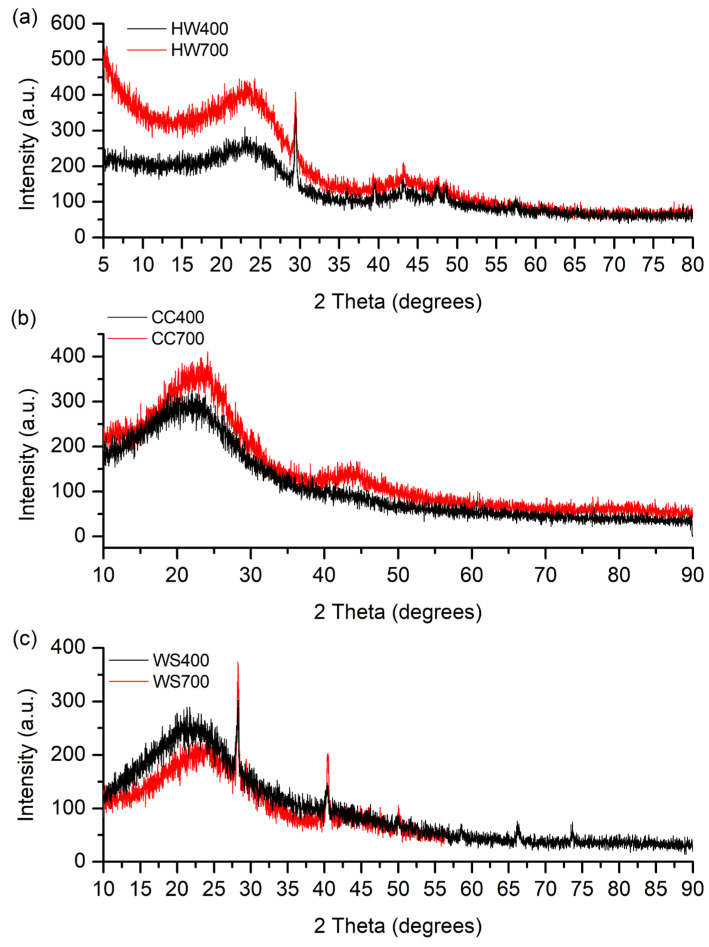
XRD diffractograms of (**a**) hardwood biochar pyrolyzed at 400 and 700 °C, (**b**) corncob biochar pyrolyzed at 400 and 700 °C, and (**c**) wheat straw biochar pyrolyzed at 400 and 700 °C.

For materials such as biochar, XRD serves as a valuable tool for identifying both amorphous and crystalline phases presented in the material [38]. In biochars, XRD patterns provide insight into both the degree of structural ordering of carbon and the presence of inorganic mineral phases derived from the original biomass ash. Increasing pyrolysis temperature typically promotes the transformation of disordered biomass carbon into more condensed aromatic domains. As Kaur et al. [15] described, cellulose in lignocellulosic biochar consists of monomers of glucose units arranged in linear chains, which form polycrystalline structures, while lignin (a three-dimensional polymer) and hemicellulose (which has both branched and straight polymer chains) are amorphous materials. The XRD patterns of biochars after pyrolysis at different temperatures are displayed in Figure 4a–c.

It can be seen from Figure 4 that the diffraction patterns for biochars originating from the same biomass are similar. The intensities of the diffractograms of HW700 and CC700 are intense in the range 2θ = 5–45° (2θ = 10–45°) compared to HW400 and CC400, while for biochar obtained from wheat straw, the intensity is higher for the one formed at a lower pyrolysis temperature (WS400). However, the most significant difference in intensity is noticeable between HW700 and HW400. This indicates a significantly higher representation of the structures that led to the formation of peaks in the specified range, specifically ordered carbon domains (such as graphitic structures), and inorganic crystalline phases (e.g., silica, carbonates, or metal oxides) [60]. The differences in intensity suggest variations in the degree of graphitization and the presence of mineral phases, which are influenced by the pyrolysis temperature and the original biomass composition [61].

In biochars obtained from hardwood and corn cobs (Figure 4a, b), two broad peaks are observed with peaks around 2θ = 23° and 2θ = 43°, with the peak at 2θ = 43° being barely visible in the CC400 biochar, i.e., of low intensity. In the biochars obtained from wheat straw (Figure 4c), a broad peak at 2θ = 23° was observed in both biochars, while the second broad peak at 2θ = 43° was observed only in WS700. Two broad diffraction peaks correspond to the presence of graphitic carbon planes (002), indicating amorphous carbon structure with randomly oriented aromatic sheets [45], and (100) structures of graphite, indicating the development of atomic order in the increasingly carbonized material [26]. According to some researchers [30,44], the broad peak at around 23° can also be attributed to cellulose residues, whose intensity gradually decreases with increasing pyrolysis temperature from 400 °C to 700 °C (except for biochars obtained from wheat straw). Kim et al. [62] compared the XRD diffractograms of raw biomass and biochars. The peaks attributed to cellulose in the XRD data of raw biomass (2θ = 16° and 2θ = 22°) were broader and less intense in biochar pyrolyzed at 300 °C, while in biochar pyrolyzed at 400 °C and 500 °C, these peaks were barely visible because the crystalline cellulose was destroyed during biochar production above 400 °C. Since the intensity of this peak in WS400 biochar is higher than in WS700, the peak can be attributed to cellulose, as was assumed in the study. The progressive intensification of these broad diffraction features with increasing temperature indicates enhanced aromatic stacking and partial graphitization of biochar carbon.

In the XRD diffraction patterns of biochars HW400 and HW700, one narrow peak was also observed, with a maximum around 2θ = 29°. The same narrow peak was also observed in biochars WS400 and WS700, as well as another narrow peak at around 2θ = 40°. Narrow peaks observed in the XRD data, especially in WS biochars, indicate the presence of mineral components, such as calcium carbonate (2θ = 29°) (JCPDS No. 47-1743), while the peak at around 40° 2θ corresponds to the (200) crystal plane in the cubic structure of quartz (JCPDS No. 46-1045) [63], corresponding to the silica in which biomass like wheat straw is rich. In biochars CC400 and CC700, no narrow peaks were observed. These crystalline mineral phases originate from the inherent inorganic fraction of the feedstock and become more prominent as organic matter is removed during pyrolysis, contributing to the ash-related properties of the biochars.

The more pronounced (002) and (100) reflections in HW700 and CC700 compared with their 400 °C counterparts are in line with previous observations that higher pyrolysis temperatures promote the development of more ordered, graphitic-like domains [60,61]. This structural ordering further supports the suitability of high-temperature biochars for applications where carbon stability and electrical or thermal properties are important.

### 2.6. Raman Spectroscopy

Raman spectroscopy has been used to further characterize the structural properties of biochar, specifically to quantify the graphitization of biochar, because Raman spectroscopy is sensitive to both crystalline and amorphous structures [15,64]. It can also be used to identify functional groups, making it complementary to FTIR [65]. However, compared to FTIR, Raman spectroscopy is more sensitive and requires minimal sample preparation, but cost is a limiting factor.

Raman spectrum characteristics include the widths, positions, and intensity ratios of the G (graphite) and D (defect) bands. By examining the overlap between the D and G bands, as well as the extensions on either side of the bands, significant details regarding the structural characteristics of the biochar can be revealed [19]. According to the studies of Gavilan et al. [66], Brubaker et al. [67], and González-Hourcade et al. [68], typical broad peaks in the Raman spectrum at 1580–1610 cm^−1^ and 1325–1380 cm^−1^ correspond to graphitic (G band) and defective (D band) structures, respectively.

**Figure 5:**
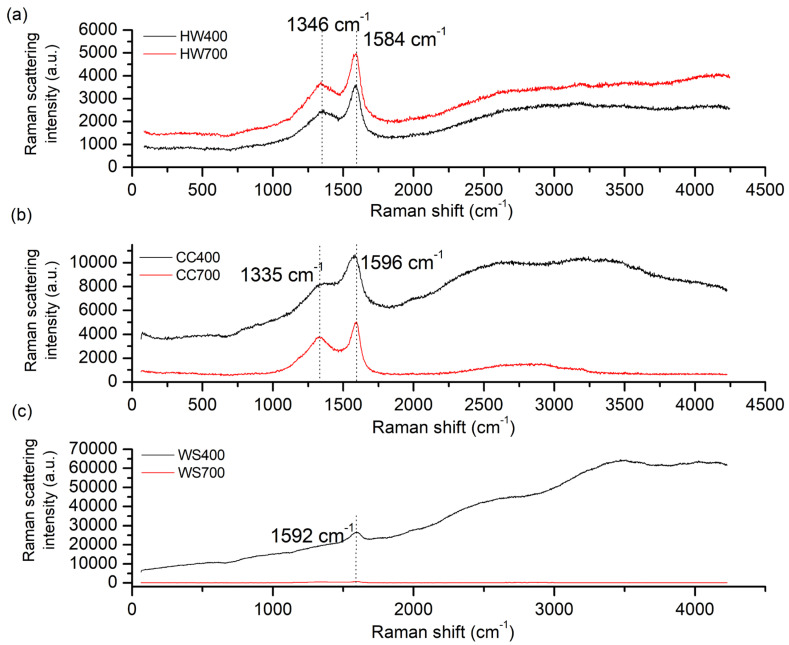
Raman spectra of (**a**) hardwood biochar pyrolyzed at 400 and 700 °C, (**b**) corncob biochar pyrolyzed at 400 and 700 °C, and (**c**) wheat straw biochar pyrolyzed at 400 and 700 °C.

Figure 5a,b shows that biochars HW400, HW700, CC400, and CC700 show a characteristic peak (D-band) at 1346 cm^−1^ (1335 cm^−1^). The D-band represents disordered or defective graphitic structures in the highly ordered carbonaceous biochar, and its intensity is related to the degree of disorder in the carbon lattice [69].

For HW400 and HW700, the Raman spectra are identical in appearance and have a pronounced D band, indicating the presence of a significant amount of disordered carbon. HW700 has a significantly higher intensity throughout the spectrum compared to HW400, suggesting that higher pyrolysis temperatures increase structural defects. For CC400 and CC700, the D band is also pronounced, but its intensity is lower for CC700, which now cannot confirm the previously mentioned relationship between structural defects and pyrolysis temperature and is consistent with the study by Yin et al. [70], who suggest that high temperatures result in the loss of functional groups, such as carbonyl and hydroxyl groups, further reducing the number of defects.

The G band, located at 1584–1596 cm^−1^ for all biochars, corresponds to in-plane vibrations of sp^2^-bonded carbon atoms in graphitic structures and may be attributed to the aromatic ring systems in the biochar [70]. Additionally, the peaks for biochars produced from the same biomass had the same shape, and the intensities behaved in the same way as for hardwood biochars.

For WS biochar, the D band is not pronounced, which may indicate a lower defect density compared to HV and CC biochars, while the G band is barely visible (especially for WS700). In general, these two biochars show a non-specific Raman spectrum, and for WS700, there is a flat line along the entire spectrum. It was not possible to calculate the I_D_/I_G_ ratio either.

The intensity ratio between the bands (I_D_/I_G_) is important to investigate the impact of the pyrolysis temperature on the degree of order/disorder of the biochar structure [71]. A comparison of the magnitude of the I_D_/I_G_ values showed that the values of HW700 (0.75), HW400 (0.70), CC700 (0.76), and CC400 (0.76) were <1, which, according to the study by Marzeddu et al. [72], indicates a high degree of graphitization. Pyrolysis temperature plays a crucial role in determining the carbon structuring of biochar [64]. If the I_D_/I_G_ ratio increases with increasing pyrolysis temperature, this indicates a greater degree of disorder and a higher density of defects within the carbon structure (as is the case with HW700 and HW400), while a decrease in the I_D_/I_G_ ratio suggests that the lattice structure of the biochar is intact, showing fewer structural defects and a higher level of graphitization [30,38]. The I_D_/I_G_ ratios below unity obtained for the HW and CC biochars are comparable to values reported for highly carbonized biochars produced at ≥600 °C [63,71,72], indicating a relatively high degree of graphitization. The slight increase in I_D_/I_G_ for HW700 compared to HW400 confirms that higher temperatures intensify structural reorganization, which is consistent with earlier Raman studies on thermally treated lignocellulosic biochars [66,67,68].

Taken together, Raman and XRD analyses confirm the progressive structural evolution of biochar from disordered lignocellulosic carbon toward more condensed aromatic domains with increasing pyrolysis temperature. This transformation is primarily driven by the degradation of polysaccharide components and the persistence of lignin-derived aromatic structures, resulting in biochars with enhanced structural order, stability, and graphitic character.

## 3. Application of Biochars Based on Results of Characterization

Biochar can be applied in various fields, depending on its diverse physicochemical characteristics [72]. In this study, a detailed characterization of biochar was investigated, which is essential for optimizing its performance and providing a comprehensive understanding, as different parameters provide a holistic view of the chemical, structural, and physical properties. Based on this, conclusions can be drawn about where the given biochars could be applied, depending on their characteristics.

The observed differences in potential applications among the produced biochars can be directly linked to the intrinsic composition of plant tissues used as feedstock. Hardwood biomass is characterized by a higher lignin content, which decomposes gradually over a broad temperature range and promotes the formation of condensed aromatic carbon structures. As a result, hardwood-derived biochars exhibit higher fixed carbon content, lower ash proportion, enhanced structural stability, and improved suitability for long-term carbon sequestration, high-temperature processes, and applications requiring chemically stable materials. In contrast, wheat straw and corn cob are richer in cellulose and hemicellulose, which decompose more readily during pyrolysis, leading to faster devolatilization, increased pore development, and higher mineral concentration in the resulting biochars. This results in higher ash content, greater surface heterogeneity, and enhanced reactivity, making these biochars particularly suitable for applications such as soil amendment, water treatment, and contaminant adsorption, where surface functionality and mineral-mediated interactions play a key role [15,16,17,20,21,22,28,29].

These application-relevant differences are further supported by specific physicochemical indicators. The alkaline nature of all biochars, which became more pronounced with increasing pyrolysis temperature, suggests their suitability for the remediation of acidic soils, which is useful in agriculture, as the introduction of such biochars can improve soil and agricultural use, where soil pH and nutrient availability can be improved [73,74]. Low O/C and (O + N)/C ratios across all samples indicate low polarizability and high aromaticity, suggesting a high potential for long-term carbon dioxide sequestration and stability, making them suitable for long-term soil application as well as CO_2_ emission reduction. The high carbon content in biochars obtained at higher pyrolysis temperatures, especially in HW700, also indicates a great potential for carbon dioxide sequestration [75]. Additionally, biochars obtained from hardwood have a high carbon content and low ash concentration, making them higher-quality biochars compared to those obtained from agricultural waste.

FTIR analysis showed that higher pyrolysis temperatures lead to increased aromatization and a decrease in oxygen-containing functional groups, which implies higher chemical stability but also the need for caution regarding the potential release of polycyclic aromatic hydrocarbons (PAHs). Therefore, biochars with simpler FTIR spectra and fewer aromatic structures, such as WS700, are preferred for environmental applications, where minimal PAH release is crucial. According to CHNS analysis, samples such as HW400 and HW700 showed low sulfur content (below the detection limit), which is especially important in combustion or soil application, where low SOx emissions are required due to strict environmental regulations. Low sulfur content in biochar helps reduce SO_2_ emissions and minimizes the risk of corrosion. Therefore, these samples are preferable for energy production and use in environmentally sensitive areas [76]. Biochars with high specific surface areas can be extremely effective as adsorbents for the reduction of contaminants in water and soil. The large specific surface area and the pronounced presence of a large number of small pores, confirmed by BET and SEM analysis, as well as the presence of polar functional groups confirmed by FTIR analysis, indicate that biochars formed at higher pyrolysis temperatures are suitable for the removal of toxins and heavy metals from polluted water [77]. Mineral components present in biochar (such as silicates and carbonates, especially in biochars from wheat straw), indicated by narrow peaks in XRD diffraction patterns, may contribute to its application in water filtration and agriculture, where it can act as a plant nutrient or improve soil structure, and it can also help in stabilizing metal(loids) [78]. EDS analysis confirmed the presence of carbonates and silicates in the analyzed biochars at concentrations above 1%.

With the rise in pyrolysis temperature, the degree of graphitization increases, and the electroconductivity of biochars increases, making them potential materials for electrodes, with appropriate modifications [79]. Biochars with high thermal stability, such as HW700 and CC700, can be used in industries that require materials resistant to high temperatures, such as catalytic reactions, energy production, and biofuel production [80]. Lower mass loss at higher temperature ranges indicates better long-term stability and less loss of useful materials at high temperatures.

Overall, the comparison of biochars produced at 400 °C and 700 °C indicates that high-temperature biochars (especially HW700 and CC700) are generally more suitable for stability-oriented applications, such as long-term carbon sequestration, high-temperature processes, and use as electrode precursors, due to their higher fixed carbon content, lower O/C and H/C ratios, greater degree of graphitization, and improved thermal resistance. In contrast, biochars produced at 400 °C retain more labile components and oxygen-containing functional groups, which may be advantageous for applications where surface reactivity and polarity are desirable, for example, in the sorption of polar contaminants or as reactive soil amendments. Thus, the optimal pyrolysis temperature within the studied range depends on the targeted functionality and end-use of the material.

The practical relevance of these findings is summarized in Table 3, which links key physicochemical properties of the produced biochars to their most suitable environmental and industrial applications.

## 4. Materials and Methods

### 4.1. Feedstock and Biochar Preparation

Biochar was produced from three biomass types: hardwood, a cob of corn, and wheat straw. Feedstocks were collected on the agricultural plot and forest region in the Autonomous Province of Vojvodina, Republic of Serbia, immediately after harvest. Wheat straw was collected after the wheat harvest in early July, corn cobs were collected following the maize harvest in September, while hardwood residues were obtained during routine forest thinning and logging operations conducted in the dormant period (late autumn–winter), all during 2023. Biochars were produced using the slow pyrolysis process described in Mutić et al. [81]. All feedstocks were manually cleaned to remove impurities, chopped into smaller pieces, and dried in an oven at 60 °C for 24 h. The dried materials were then subjected to slow pyrolysis in a Nabertherm furnace (Lilienthal, Germany) (temperatures from 30 to 3000 °C, Germany). The pyrolizer was closed (to prevent oxygen flow), and the temperature was increased to the target value (400 °C or 700 °C) at a heating rate of 10 °C/min. Pyrolysis was performed under an inert argon atmosphere (80 L/h) to ensure oxygen-free conditions throughout the process. Once the target temperature was reached, samples were maintained at that temperature for approximately 1 h. After completion of pyrolysis, the material was allowed to cool naturally to room temperature inside the closed furnace. The resulting biochars were labelled HW400, HW700, CC400, CC700, WS400, and WS700 to denote feedstock type and pyrolysis temperature.

The selected raw materials—hardwood, wheat straw, and corn cob—were chosen to represent diverse lignocellulosic biomass types with different chemical compositions. Hardwood belongs to woody waste biomass, and wheat straw and corn cob to non-woody waste biomass [15]. Hardwood, rich in lignin, offers a thermally stable structure, while wheat straw and corn cob contain higher amounts of cellulose and hemicellulose, which decompose more readily during pyrolysis. These differences enable a comparative evaluation of how feedstock composition affects biochar properties. Additionally, all three are widely available agricultural or forestry residues, making them relevant for sustainable biochar production with potential environmental and agricultural applications.

### 4.2. Biochar Characterization

Characterization techniques included Fourier transform infrared spectroscopy (FTIR), the Brunauer–Emmett–Teller method (BET), X-ray diffraction (XRD), Raman spectroscopy, thermo-gravimetric analysis (TGA), scanning electron microscopy (SEM), energy-dispersive X-ray spectroscopy (EDS) and CHNS elemental analysis, pH measurement, and determination of moisture, ash, volatile matter, and fixed carbon contents.

All measurements were performed in triplicate (n = 3), and the reported values represent mean values ± standard deviation.

### 4.3. pH

The pH of the biochars was measured in a 1:10 solid/solution ratio of deionized water and mixed thoroughly using a rotary shaker at 50–60 rpm for 1 h. All the samples were analyzed in triplicate, and average values were reported.

### 4.4. Moisture, Volatile Matter, and Ash Content

Moisture, volatile matter, and ash content were determined according to the international standard procedure ASTM D1762–84 [82]. About 1 g of all six materials was weighed in duplicate in pre-weighed crucibles and placed in an oven heated to 105 °C. The samples were dried to a constant mass. The dried samples were left in a desiccator for 1 h and weighed. The difference in mass loss equaled the moisture content. The crucibles used for moisture determination, containing the sample, were heated as follows: with the oven door open, 2 min at the outer edge of the oven (300 °C), then 3 min at the edge of the oven (500 °C). They were then moved to the back of the oven for 6 min with the door closed. After 1 h in the desiccator, the samples were weighed. The difference in mass loss equaled the volatile content. After that, the crucibles were placed in an annealing furnace and annealed to a constant mass at 550 °C (not less than 6 h). The difference in mass loss was considered the ash content. Fixed carbon (%) was calculated using the equation %fixed carbon = 100 − (%moisture + %ash + %volatile matter).

### 4.5. CHNS

Elemental compositions (C, H, N, and S) were determined using an Elementar Vario Macro Cube analyzer (Elementar Analysensysteme GmbH, Langenselbold, Germany). Samples were combusted in an oxygen atmosphere at 1200 °C, and the resulting gases were quantified using a thermal conductivity detector (TCD). The instrument accuracy was 0.1%.

### 4.6. FTIR

The identification of functional groups present on the surface of the material of interest was achieved by Fourier transform infrared spectroscopy (FTIR spectroscopy, “Nicolet Nekus 670, Thermo Fisher Scientific, Waltham, MA, USA”). Sample preparation consisted of the formation of KBr-based tablets, while spectrum reading was performed in a range from 4000 cm^−1^ to 400 cm^−1^ in the diffuse reflection mode, at a resolution of 4 cm^−1^.

### 4.7. SEM

The surface morphology and structural features of the biochars were examined using a Thermo Fisher Scientific Apreo C scanning electron microscope (Waltham, MA, USA).

### 4.8. BET

The specific surface area of the biochars was determined using nitrogen adsorption at 77 K on a QuantaChrome Nova 2000 (Quantachrome Instruments, Boynton Beach, FL, USA) surface area analyzer. Prior to measurement, all samples were degassed at 423 K for 1 h to remove adsorbed moisture and volatile contaminants. The Brunauer–Emmett–Teller (BET) method was applied to the adsorption isotherm to calculate the specific surface area.

### 4.9. XRD

Powder X-ray diffractogram (XRD) patterns were recorded using a Rigaku Miniflex (The Woodlands, TX, USA) 2 unit equipped with a Cu Kα radiation source (4°/min resolution, 2θ = 4–80° (2θ = 10–80°)) to assess the crystalline properties of the materials.

### 4.10. Raman

To assess the degree of graphitization, Raman characterization was performed at an excitation wavelength of 532 nm and a nominal laser power of 12.5 mW (Senterra Bruker Optik GmbH, Ettlingen, Germany). The spectral resolution was set to ca. 3–5 cm^−1^, and the interferometer resolution was 1.5 cm^−1^.

### 4.11. TGA

Thermogravimetry–differential thermogravimetry (TG–dTG) measurements were conducted using a TA Q500 TGA instrument (TA Instruments, New Castle, PA, USA), to understand decomposition behavior and long-term stability. The measurement conditions were at a heating rate of 5 °C/min, in N2 or air atmosphere, with a measurement range from 25 to 1000 °C.

### 4.12. EDS

The sample elemental composition was determined using X-ray electron diffraction spectroscopy (EDS) with built-in scanning electron microscopy. The microscope was operated at a 40 nA current and 20 kV acceleration voltage.

By combining data from CHNS elemental analysis and oxygen percentage obtained from EDS analysis, the atomic ratios H/C, O/C, and (N + O)/C were calculated.

### 4.13. Higher Heating Values (HHVs)

The higher heating values (HHVs) of the produced biochars were estimated using an empirical correlation based on elemental composition, as proposed by Palamanit et al. [83]. The HHV (MJ kg^−1^) was calculated according to the following equation:

$${\mathrm{HHV}\;(\mathrm{MJ}\;\mathrm{kg}}^{-1})=0.341C+1.322H-0.12O-0.12N+0.0686S-0.0153Ash$$

where *C*, *H*, *N*, *S*, *O*, and *Ash* represent the mass percentages (wt.%) of carbon, hydrogen, nitrogen, sulfur, oxygen, and ash, respectively.

## 5. Conclusions

Among all biochars analyzed and characterized, those produced at higher pyrolysis temperatures (HW700, CC700, and WS700) generally exhibited higher pH, carbon, and ash content and specific surface area compared to HW400, CC400, and WS400. All samples showed H/C molar ratios < 1.5 and low O/C molar ratios, indicating highly aromatic, low-polarity structures with an estimated half-life exceeding 1000 years. Biochar produced at lower temperatures exhibited higher (O + N)/C ratios, reflecting a greater abundance of polar functional groups and enhanced surface reactivity compared to high-temperature biochars.

FTIR analysis confirmed progressive removal of oxygen-containing functional groups and increased aromatization with increasing pyrolysis temperature. SEM images revealed increased porosity with increasing pyrolysis temperature, characterized by the development of cracks and well-defined pores. EDS and CHNS analysis showed that all biochars are predominantly composed of carbon and oxygen, while other elements are present in minor amounts (<1%). XRD and Raman analysis indicated a transition toward more ordered and graphitized carbon structures at higher temperatures, with inorganic phases such as SiO_2_ and CaCO_3_ contributing to the mineral content, particularly in wheat straw biochars. Thermogravimetric analysis showed that thermal behavior was primarily feedstock-dependent: hardwood and corn cob biochars exhibited similar mass losses at both pyrolysis temperatures, whereas wheat straw biochar produced at 700 °C showed lower overall mass loss and higher thermal resistance.

Overall, the comprehensive characterization of biochars from hardwood, corn cob, and wheat straw produced at 400 °C and 700 °C demonstrates their suitability for diverse environmental and industrial applications, with optimal selection governed by feedstock composition and target functionality.

## Figures and Tables

**Table 1:** Characterization of biochars.

Parameters	HW400	HW700	CC400	CC700	WS400	WS700
pH	9.06 ± 0.01	9.40 ± 0.26	7.94 ± 0.13	9.45 ± 0.10	9.21 ± 0.02	10.3 ± 0.20
Ash (% *w*/*w*)	3.25 ± 0.14	5.98 ± 1.12	9.4 ± 1.45	16.2 ± 2.24	15.2 ± 1.05	24.8 ± 2.56
Moisture (% *w*/*w*)	2.81 ± 0.11	3.03 ± 0.13	4.09 ± 0.21	2.81 ± 0.12	4.28 ± 0.32	5.25 ± 0.21
Volatile matter (% *w*/*w*)	41.4 ± 5.12	24.8 ± 3.15	56.1 ± 6.25	24.1 ± 4.12	54.1 ± 4.15	10.2 ± 3.19
Fixed carbon **^a^** (% *w*/*w*)	52.5 ± 1.79	66.2 ± 1.47	30.4 ± 2.64	56.9 ± 2.16	26.4 ± 1.84	59.7 ± 1.99
C (% *w*/*w*)	82.4 ± 1.47	89.8 ± 2.47	71.9 ± 0.12	82.9 ± 0.42	66.7 ± 2.38	66.3 ± 2.23
H (% *w*/*w*)	0.40 ± 0.07	0.90 ± 0.05	4.41 ± 0.14	1.61 ± 0.08	4.30 ± 0.25	1.66 ± 0.01
N (% *w*/*w*)	0.42 ± 0.08	0.40 ± 0.04	0.64 ± 0.12	0.33 ± 0.01	1.41 ± 0.60	0.94 ± 0.07
S (% *w*/*w*)	<0.03	<0.03	4.09 ± 0.14	4.36 ± 0.82	2.84 ± 0.72	2.01 ± 0.32
O (% *w*/*w*)	12.5 ± 0.19	7.79 ± 0.50	5.21 ± 0.28	1.42 ± 1.34	8.47 ± 7.51	6.55 ± 2.53
O/C (molar ratio)	0.11	0.07	0.05	0.01	0.09	0.07
H/C (molar ratio)	0.06	0.12	0.73	0.23	0.77	0.30
(O + N)/C (molar ratio)	0.12	0.07	0.06	0.02	0.11	0.09
HHV (MJ/kg)	27.0 ± 0.51	30.7 ± 0.8	29.8 ± 0.19	30.2 ± 0.25	27.2 ± 1.26	23.7 ± 0.82

Values ± standard deviation. ^a^ Determined by difference. Mean values ± standard deviation. **Note**: Proximate analysis (moisture, volatile matter, ash, and fixed carbon) and ultimate analysis (CHNS) were performed using independent analytical methods. Fixed carbon was calculated as 100 − (moisture + ash + volatile matter), while oxygen was estimated from SEM–EDS data. As these analyses are not designed to be additive, the sum C + H + N + S + O + ash is not expected to equal 100%.

**Table 2:** Cause of loss and percentage of loss of weight of biochar at certain temperature ranges.

TG	Temperature Interval	Biochars					
		HW400	HW700	CC400	CC700	WS400	WS700
Total Weight Loss (%)	20–800 °C	96.0	96.5	95.5	95.1	89.3	81.8
Moisture	20–105 °C	3.37	3.63	5.16	6.40	5.33	7.17
Moisture and very labile OM	105–200 °C	0.539	0.342	0.388	0.929	0.813	1.420
Labile OM	200–400 °C	7.52	6.39	34.9	11.9	41.2	28.5
Intermediate OM	400–600 °C	84.5	85.6	54.2	74.6	40.2	42.6
Recalcitrant OM	600–800 °C	0.129	0.542	0.775	1.26	1.79	2.04

**Table 3:** Recommended applications of biochars based on their physicochemical properties.

Biochar	Key Physico-Chemical Characteristics	Recommended Applications	Explanation
**HW700**	High aromaticity (low H/C and O/C ratios); high specific surface area; very low sulfur content; high graphitization	Long-term carbon sequestration; adsorption of organic pollutants; high-temperature industrial applications (catalysis, energy systems)	High structural stability and durability; large surface area enhances adsorption; low sulfur minimizes SOx emissions
**CC700**	Strong development of microporosity; high specific surface area	Heavy metal adsorption in water and soil; catalytic applications; water treatment	Developed pore structure promotes adsorption and catalytic activity
**WS700**	High ash content; strong alkalinity (pH 10.3); presence of mineral phases (SiO_2_ and CaCO_3_); good thermal stability	Soil remediation and pH correction; metal(loid) immobilization; agricultural soil amendment	Mineral-rich composition improves soil properties and stabilizes contaminants
**HW400**	Higher content of oxygen- and nitrogen-containing functional groups; moderate surface area	Nutrient retention in soils; short- to medium-term soil amendment; adsorption of polar compounds	Abundance of polar functional groups increases reactivity and cation exchange capacity
**CC400**	Pronounced surface functionalization; lower specific surface area than CC700	Soil fertility enhancement; reactive environmental applications	Retains labile functional groups that enhance chemical interactions
**WS400**	High surface functionalization; lowest specific surface area	Short-term soil amendment; nutrient retention; applications where surface polarity is required	Enhanced polarity and reactivity compensate for lower porosity

## Data Availability

The original contributions presented in this study are included in the article. Further inquiries can be directed to the corresponding author.
